# Hyperspectral Imaging in Environmental Monitoring: A Review of Recent Developments and Technological Advances in Compact Field Deployable Systems

**DOI:** 10.3390/s19143071

**Published:** 2019-07-11

**Authors:** Mary B. Stuart, Andrew J. S. McGonigle, Jon R. Willmott

**Affiliations:** 1 Department of Electronic and Electrical Engineering, University of Sheffield, Sheffield S1 4DE, UK; 2 Department of Geography, University of Sheffield, Sheffield S10 2TN, UK; 3 School of Geosciences, The University of Sydney, Sydney, NSW 2006, Australia; 4 Faculty of Health, Engineering and Sciences, University of Southern Queensland, Toowoomba, QLD 4350, Australia

**Keywords:** hyperspectral, environmental monitoring, miniaturization, low-cost, field deployable

## Abstract

The development and uptake of field deployable hyperspectral imaging systems within environmental monitoring represents an exciting and innovative development that could revolutionize a number of sensing applications in the coming decades. In this article we focus on the successful miniaturization and improved portability of hyperspectral sensors, covering their application both from aerial and ground-based platforms in a number of environmental application areas, highlighting in particular the recent implementation of low-cost consumer technology in this context. At present, these devices largely complement existing monitoring approaches, however, as technology continues to improve, these units are moving towards reaching a standard suitable for stand-alone monitoring in the not too distant future. As these low-cost and light-weight devices are already producing scientific grade results, they now have the potential to significantly improve accessibility to hyperspectral monitoring technology, as well as vastly proliferating acquisition of such datasets.

## 1. Introduction

Over the past three decades, hyperspectral imaging has emerged as an effective tool for a variety of applications ranging from remote sensing of the Earth’s surface [1,2,3], to art conservation and archaeology [4,5,6]. Whilst spectral imaging with multispectral sensors has been achieved since the late 1960s [7], recent advances in the spectral and spatial resolution of sensors has opened-the-door to more detailed scene analysis with hyperspectral imaging [2,8]. 

Hyperspectral images are characterized by both their spatial and spectral resolution [9,10], e.g., with two spatial dimensions (S_x_ and S_y_) and one spectral dimension (S_λ_). The spatial resolution measures the geometric relationship between the image pixels, while the spectral resolution determines the variations in illumination within the image pixels as a function of wavelength [3]. These data are represented in the form of a 3-Dimensional hyperspectral data cube [2,3], where each “slice” of this data cube along S_λ_, represents a specific band from the electromagnetic spectrum [3].

Initially developed for remote sensing applications [4,11], hyperspectral imaging sensors typically acquire images across hundreds of narrow spectral bands within the visible, Near Infrared (NIR), and Mid Infrared (MIR) segments of the electromagnetic spectrum [3,11]. This enables the construction of an almost continuous reflectance spectrum for each pixel in a scene which, in turn, allows for the in-depth spectral examination of scene features that would be rather less perceptible with the coarser bandwidths of multispectral scanners [1,7,8,12]. This recent development in sensor technologies has led to the uptake of hyperspectral imaging methods across a wide variety of disciplines, opening new possibilities for measuring and monitoring multiple aspects of our environment [8]. 

In recent years, there has been a considerable uptake of field deployable hyperspectral imaging within the discipline of environmental monitoring [13,14]. This is an exciting, and potentially revolutionary, development that could result in substantial future alterations to existing monitoring methods and sensing modalities, involving capture of higher quality data [15]. It is, therefore, highly timely and important to capture the current state-of-play in this field at this juncture, which is the motivation behind the development of this article. Here, we provide a review of current hyperspectral technologies and their integration into the environmental monitoring field, with a particular focus on the successful miniaturization and improved portability of these sensors, as well as highlighting the recent move towards the implementation of low-cost consumer market technology. Recent developments are discussed, focusing on key examples across a variety of environmental disciplines, emphasizing the significant enhancements these developments have made to data acquisition for both ground-based and aerial deployments. 

The focus of this article is to review the current progress in low-cost, field deployable hyperspectral devices for use within environmental monitoring applications. Our research methodology, therefore, focused on the following search terms; “low-cost”, “miniaturization”/“miniaturized”, “hyperspectral”, and “environmental monitoring”. These terms were used to search three online scientific citation indexing services (Web of Science, Scopus, and Google Scholar) in order to obtain the articles that make up this review. These databases were used to elucidate the key researchers that have published in the aforementioned categories of hyperspectral imaging. This allowed us to see the current leading edge in the field, by seeing where the research leaders were most active. The second strand to our method was to build an understanding of the hyperspectral modalities that we believe is comprehensive. These models are described below and we have conjoined our understanding of these modalities with our review of the state-of-the-art. As our focus is field deployable devices, articles pertaining to satellite-based applications, such as CubeSat, have largely been excluded from this work. Whilst these satellite-based applications often represent low-cost, miniaturized devices, they are not inherently field deployable and, therefore, do not fit the narrative of this article. In this review, we have provided a comprehensive repository for information on the different design approaches to hyperspectral imaging for field-deployable systems; by whom the leading research is being conducted; the nature of the research; and our interpretation of how the research fits within the overall research field.

## 2. Sensor Types

**Figure 1:**
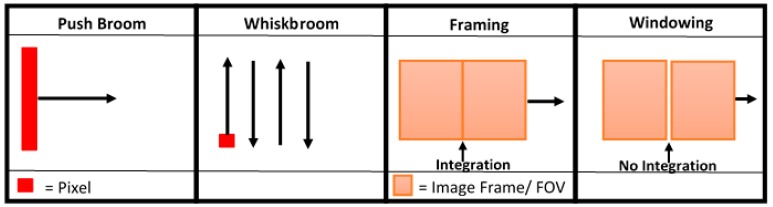
Image capturing techniques for each sensor type. Note the different methods of image formation; from the pixel-based capture of Push Broom and Whiskbroom scanners, to the 2-Dimensional comprehensive image capture of Framing and Windowing instruments. This highlights the potential issues related to image distortion resulting from the rotation of the optics in the pixel-based instruments, as mentioned above.

There are a number of different approaches to hyperspectral imaging and, as such, a variety of sensor types are available (Figure 1) [10]. Typically, sensors are characterized by the arrangement and/or the number of spectral bands involved in the instrumental architecture [10,16], as well as the applied image capture method. Push broom sensors have been traditionally used for large airborne imaging applications and have recently been successfully miniaturized for use within UAV (unmanned aerial vehicle) systems [10,17,18]. This push broom measurement approach is favored due to its high spatial and spectral resolution [19], however, this image acquisition method, whereby a line of spectral information per exposure is recorded [10,20], can cause difficulties in post-processing [10]. Similarly, whiskbroom sensors, which image a single pixel or spatial location at a time [21,22], using a rotating mirror to sweep a scan line perpendicular to the direction of the sensor platform’s movement [21,22,23], are affected by the same issues [21]. Furthermore, whiskbroom sensors provide inherently slower frame rates than Push Broom units, resulting in lengthier data acquisition periods where all other things are equal [21,24]. Another disadvantage is that the rotation of the optics can result in spatial distortions in the image outputs [25]. However, recent work reported by Uto et al. [22], has demonstrated the pioneering of low-cost whiskbroom image collection suitable for UAV deployment. 

Alternatively, framing instruments (Figure 1) can capture scenes through 2-Dimensional images with additional optics that focus on either an individual wavelength or wavelength bands using tunable filters, such as framing band pass filters translated across the spectrum [25]. The design of such sensors is significantly simpler than those of push broom and whiskbroom sensors [21,26], however, the use of spectral filtering substantially reduces the intensity of light captured at the sensor, limiting signal to noise [25]. Windowing instruments also employ a 2-Dimensional Field of View (FOV) that moves across a scene in a continuous fashion [27]. However, instruments that utilize this image capture approach acquire a distinct exposure each time the FOV moves forward, with no integration between exposures [27].

The literature highlights that although there can be significant variation caused by slit width, lens focal length, and integration time [10], Push Broom sensors, at present, offer a better combination of spatial and spectral resolution. Push Broom sensors are typically more stable than Whiskbroom sensors due to the line-by-line image acquisition process, therefore, confining potential data misalignments to between lines rather than between individual pixels [19]. Furthermore, they often have a significantly greater spectral resolution, for example Jaud et al. [26], reports a spectral resolution of 1.85 nm for their Push Broom device. Framing and Windowing devices are often limited due to the filtering of spectral bands, resulting in spectral resolutions of >5 nm being more common for these devices [10,27]. High spatial resolution is also easier to achieve with current Push Broom devices as miniaturization allows for them to be deployed on more maneuverable, light-weight devices, for example, a number of studies highlight successful image acquisitions with spatial resolutions of less than 10 cm [26], with Lucieer et al. [17], and Malenovský et al. [18], achieving a spatial resolution of 4 cm with UAV based deployments. Framing and Windowing devices are currently limited due to their typically larger size, making Push Broom sensors significantly more compatible to light-weight, miniaturized sensing applications at present.

**Figure 2:**
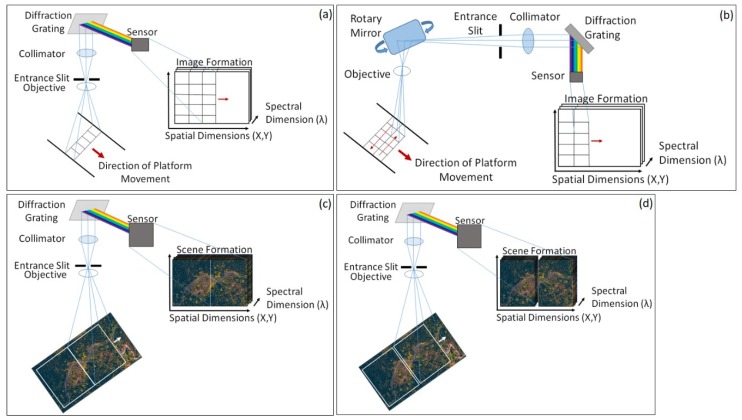
Typical schematic designs for each sensor type. (**a**) Push Broom sensor; (**b**) Whiskbroom sensor; (**c**) Framing sensor; (**d**) Windowing sensor. Note the lack of integration between image tiles for Windowing sensor designs. Image not to scale.

Although several of these sensor designs (Figure 2) have been successfully miniaturized, making them suitable for light-weight aerial remote sensing, they do not currently contain any internal georeferencing data and, therefore, require the addition of external (e.g., GPS receiver) devices to record this information if it is required [3,10]. Whilst this does not particularly effect traditional remote sensing and ground-based imaging methods, it can become problematic when designing effective UAV integrated payloads [10,21]. Each of these sensor designs has its advantages, depending on the parameters of the proposed application, however, the push broom design has been the most popular, particularly within the field of light-weight UAV image acquisition [19]. Whilst these sensor implementations can involve distortions within the acquired data, they currently outperform full-frame image capturing approaches as the latter systems currently require a compromise between spatial coverage, spatial resolution and spectral resolution [10,26]. However, as interest and demand within this area continues to grow [8,19], significant advances in compact sensor designs, including the incorporation of linear variable filters, can be anticipated in the future.

## 3. Technological Developments and Associated Complexities

Currently, hyperspectral imaging is generally performed by satellite or aircraft platforms [20,26,28], with recent advances in airborne and spaceborne technologies providing end users with rich spectral, spatial, and temporal information [1,2]. As such, hyperspectral imaging has been well established in the remote sensing community, with large-scale uptake across many different domains [4,10]. Furthermore, the recent development of CubeSat miniature satellites, such as HyperCube [29], shows significant potential for future development of light-weight, low-cost spaceborne image acquisition [30,31,32]. However, whilst these sensors enable the analysis of extensive areas of the Earth’s surface, providing large-scale datasets with long time series [26], they are often constrained by factors outside the users’ control, such as cloud coverage and spatial resolution [1,19,26]. Furthermore, manned aerial surveys operated on an on-demand basis can be rather expensive and somewhat reliant on favorable meteorological conditions [11]. As a result, these drawbacks significantly limit the suitability of these measurement types for many smaller scale, local applications.

Jaud et al. [26], highlights this sizable gap between the small-scale, fine resolution outputs of field surveys and the comparatively coarse resolution provided by satellite and aerial sensors. However, the development of UAV platforms over the last decade has enabled the development of an intermediary protocol, in the form of UAV integrated hyperspectral sensing [8,11,20,33]. These UAV based platforms provide greater flexibility than traditional sensing methods, permitting the user to vary parameters such as survey size and flight altitude [12,26], in a manner tailored to the proposed application. Additionally, due to their typically small size and low weight they can be easily, and readily, stored and deployed [33,34]. A number of UAV integrated hyperspectral sensors have been tested in recent years within a variety of different fields; Habib et al. [20], present a low-cost UAV integrated hyperspectral scanner applied to the field of precision agriculture. Their multi-rotor system proved successful, providing detailed imagery of the survey area, however, difficulties arose during the georectification process, with the accurate generation of georeferenced products proving difficult to establish [20]. Similarly, Jaud et al. [26], experienced complications during the line-by-line georectification and referencing required of their push broom, multi-rotor UAV sensor acquisitions, with the push broom image formation process leading to a major source of complexity during the geometrical correction step [26]. 

### Georectification Difficulties

Due to the light-weight nature of multi-rotor UAV systems they generate substantial high frequency vibrations and can perform faster trajectory changes than larger platforms, therefore, these systems require fast, accurate proprioceptive sensors to enable accurate logging of altitude and position [26,35]. Mozgeris et al. [36], directly compared the results obtained from a UAV based hyperspectral imaging camera and a similar sensor based within a manned, fixed wing, ultra-light aircraft in the context of precision agriculture monitoring at a site in Lithuania. They determined that the manned aircraft sensor outperformed the UAV based device in terms of the quality of output data as a function of cost. A key factor in this was the higher relative accuracy of georeferencing in the case of the manned deployment, which the higher spatial resolution coverage of the UAV sensor was not sufficient to counteract [36]. Conversely, Freitas et al. [19], present a direct georectification method applied on their fixed wing UAV based sensor, which substantially improved the accuracy of target georeferencing. Whilst they still experienced difficulties due to the nature of push broom image acquisition, the results obtained suggest that reliable acquisition of accurately georeferenced data using a UAV based sensor is now possible. 

A number of studies have circumvented these georectification issues simply by implementing ground-based data acquisition protocols [37], however, the obtained images can still be affected by other factors, such as, variable meteorological conditions [8]. Indeed, this issue can affect both ground-based and aerial hyperspectral imaging [24,37]. Variations in illumination, in particular, during the study period can have a significant effect on the captured data, introducing apparent changes in captured spectra unrelated to changes in the scene surface covering [8,20,24,37]. However, the effect of these variations can be minimized by recording trends in illumination in parallel with the image capture [8], which can be used to calibrate the hyperspectral image data acquired during these periods [3,19,24].

The demand for smaller and lighter hyperspectral imaging sensors continues to grow, with the application of UAV integrated sensors being one of the most rapidly developing areas of remote sensing technology [8,12]. The desire to reduce the physical size of these sensor systems whilst maintaining the data quality available from larger units is an aspiration in both aerial and ground-based sensing configurations [12,37]. With the advent of widely available 3D printing services [38,39], and the continued development of sensors for both scientific and commercial purposes [11], the opportunities to pioneer units specifically tailored to desired application areas have never been greater. Whilst at present, push broom and whiskbroom sensors are subject to limitations in temporal resolution, associated with the georectification process, there are considerable ongoing improvements in accurate direct and indirect georectification methods [19,26]. In general, the continued development of more compact, light-weight devices creates the opportunity for imaging surveys with high spatial and spectral resolutions, delivering added flexibility in the acquisition parameters [11,26,37].

## 4. Applications within Environmental Monitoring

As highlighted in the sections above there is considerable potential for, and progress towards, compact, field portable hyperspectral imaging sensors for a variety of environmental monitoring applications. With the additional benefits of integrating low-cost, high quality consumer market components, there is now a significant opportunity to make hyperspectral imaging more common within environmental monitoring. There has, therefore, been a wide variety of devices developed for sensing applications across these conditions. Due to the significant variations between these settings the devices required can differ substantially in terms to size, weight, and robustness, to name a few factors. This section will discuss developments across these contrasting environments, concentrating on some key examples, to illustrate the current state-of-the art in the field. Within this section the term “low-cost” is used to refer to hyperspectral devices assembled, often “in house”, from mass produced components allowing for the overall build costs to be significantly lower than that of commercial, scientific grade instruments. 

### 4.1. UAV Based Applications

#### 4.1.1. Agricultural and Natural Vegetation Monitoring

As discussed above, the development of light-weight, and low-cost, UAV compatible sensors is a rapidly expanding area of research resulting in significant developments across a wide range of environmental monitoring applications. Whilst there are potential issues relating to the georectification process [20,26,40], the benefits related to improvements in spatial resolution and reduced fieldwork costs are substantial. The monitoring of vegetation across both natural and agricultural environments is a particular area of environmental monitoring that has benefitted from the advances in miniaturization and cost reduction of hyperspectral technologies [14,41], allowing for precise, in-depth monitoring and data collection to be accomplished even in the most inaccessible locations. The light-weight sensors that have been developed to date show significant potential in their application for close-range environmental monitoring [14], with the introduction of devices for monitoring vegetation health receiving particular attention [41,42,43,44]. The continued monitoring of these environments with hyperspectral technologies is of considerable importance. Due to the spectral resolution of these devices it is possible to observe areas of vegetation stress, such as water stress or potential pest outbreaks, before they become visible to the naked eye. This is done through the examination of pigments, such as Chlorophyll, that will vary in quantity depending on the health of the vegetation, subsequently effecting its spectral response. In the initial stages of vegetation stress these changes can be subtle and, therefore, best recognized with hyperspectral imaging. This, in turn, allows for any potential issues to be resolved or minimized before significant damage can be done.

Traditional monitoring methods for both agricultural and natural vegetation typically require time consuming direct measurements or the use of spaceborne sensors [45,46], with limitations in spatial resolution in respect of the latter [47,48]. The introduction of UAV based hyperspectral sensors creates the opportunity to acquire accurate, close-range data that do not require the complex processing typical of satellite and high altitude airborne systems. Indeed, these UAV deployments aim to deliver data in an intermediary format, which provides both the satellite-based benefits of spatial coverage as well as the spatial resolution afforded from ground-based deployments [15,49,50]. In particular, Garzonio et al. [15] present a multi-rotor UAV equipped with a cost-effective hyperspectral sensor capable of detecting wavelengths within the visible and NIR (350–1000 nm) for a variety of vegetation monitoring applications. Due to the multi-rotor design, the device presented was capable of both transect and fixed target measurements, allowing it to be utilised for a variety of scenarios. Furthermore, it provided a systematic and rapid method of high quality data collection, suitable for relatively inaccessible locations, such as dense vegetation forests and forest canopies, allowing large, high resolution datasets to be collected with relative ease [15]. However, despite overcoming issues related to in-flight mechanical vibration of the sensor, the spectral resolution and signal to noise ratio of the device were not optimal to capture all of the desired measurements, with particular problems related to the capture of sun-induced fluorescence data [15].

Similarly, Näsi et al. [14] deployed such technology for monitoring insect damage across urban forests. Their low-cost sensor enabled analysis at an individual tree level, providing a new level of specificity in forest health management practices [14,43]. Whilst such detailed spatial resolution has been achieved by a few studies in the past, such as Minařík and Langhammer [51], and Dash et al. [52], they pertain, solely, to multispectral approaches. This hyperspectral unit [14] performed well, however, difficulties were encountered related to temporal illumination changes during the data acquisition [14]. As highlighted above, this is a potential issue that is generic to hyperspectral imaging from most airborne, and ground-based, devices [8,19], and is, therefore, not a result of the low-cost of this device, but simply a factor that requires attention during extended data acquisitions. A method that provides the simultaneous monitoring of illumination change and data acquisition, and/or reference panel measurements would help to minimize these issues in future work [8,53]. Despite these minor setbacks, the development of these new, easy to use technologies could have significant benefits for monitoring of both urban and rural forest health, with these low-cost units enabling far wider sensor proliferation than possible hitherto, with the more expensive previously applied instrumentation. This in turn could lead to significant benefits in terms of avoidance of future pest outbreaks and the potential resulting forest losses [14,54]. 

A number of other studies have utilised similar UAV based techniques for the monitoring of agricultural vegetation [55,56], and soil quality [13,57], producing accurate, high spatial resolution results, further emphasizing the wide-ranging usability of these designs. However, there remain limitations related to the weight and power supply of these devices, with heavier payloads having a negative effect on the potential duration of aerial surveys [15]. Whilst this is limiting the practical utilization of these devices at present, as technologies continue to be miniaturized and UAVs themselves advance, survey flight times will become proportionately longer in the future [12]. 

#### 4.1.2. Extreme Environment Monitoring

A particular benefit of the continued development of these devices is that they allow non-destructive data acquisition, which is of considerable importance for highly sensitive and/or protected environments, which are often a key focus of environmental monitoring research and operations. Moreover, they also enable the acquisition of high spatial resolution data from locations where ground-based field surveys would prove impractical or hazardous. Key examples here include glacial and ice sheet regions, which have been host to considerable UAV based monitoring, for example Crocker et al. [58], Hugenholtz et al. [59], Rippin et al. [60], and Ryan et al. [61]. However, work in this domain to date has been largely restricted to multispectral and/or photogrammetry-based data acquisitions, with hyperspectral monitoring being mostly confined to spaceborne observations [62]. The addition of field portable hyperspectral sensing to glacial settings will provide a significant improvement to current datasets, such as the identification of supraglacial debris composition in otherwise difficult to access locations [63]. Application of UAV based hyperspectral image capture in the cryosphere is likely to be a highly promising future area of research.

#### 4.1.3. Pollution and Particulate Monitoring

Inland water quality and pollution monitoring with hyperspectral sensors, has only recently involved a move away from purely spaceborne imaging methods [64,65]. This change has been largely driven by the limitations of satellite-based remote sensing as the spatial resolution provided by most such sensors is somewhat limited, without substantial pixel mixing [64,65,66]. Hyperspectral sensors used to monitor these environments provide high resolution optical data that allows for the simultaneous detection and monitoring of air and water quality. This provides an extensive and accurate means of pin-pointing potential pollution outbreaks and/or monitoring the quality of freshwater sources across relatively large areas. Although the majority of recently developed sensors within these fields remain aircraft based [64,67,68,69,70,71], with the advantage of coverage of larger survey areas than typically possible with UAVs, a number of pioneering optical sensors for pollution and particulate monitoring are beginning to emerge. These new devices are providing significant improvements to current monitoring techniques with the introduction of UAV based [72,73], and lower cost portable [74], approaches. The promising success rates of these new devices are providing significant improvements to our understanding of particulate pollutants [75], whilst also highlighting the substantial scope for further development and integration of UAV based hyperspectral sensor systems to this field. 

### 4.2. Hand-Held and Ground-Based Device Applications

**Figure 3:**
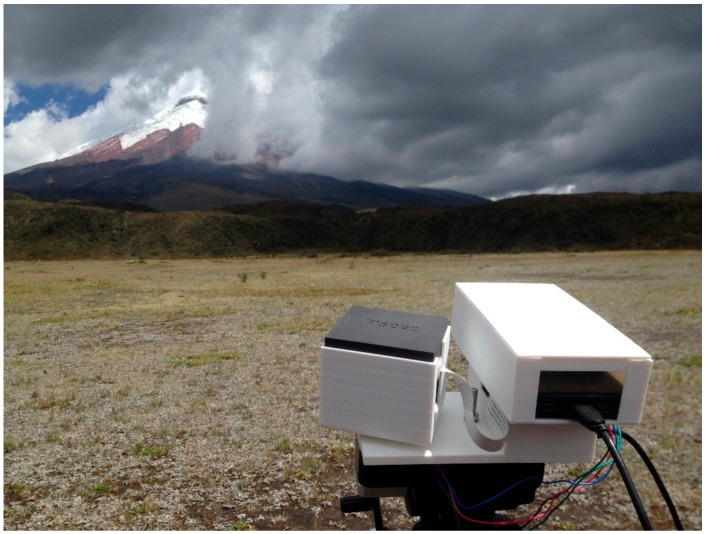
Compact UV hyperspectral imager measuring Sulphur Dioxide release from Cotopaxi volcano, Ecuador.

Whilst the majority of hyperspectral sensing measurements have been achieved from airborne platforms, there have also been significant developments in hand-held and ground-based hyperspectral sensing in recent years [47,48]. These devices are typically relatively light-weight and field portable, (Figure 3) making them of significant benefit to a variety of small-scale fieldwork-based studies. However, as this hardware is not subjected to the stringent payload requirements of UAV compatible devices, there are relaxed tolerances with regards to weight, bulk, and power supply. A variety of miniaturized hand-held sensors have been developed for several applications, with a degree of device commercialization implicit in this activity [76,77]. In particular, Shan et al. [78] have developed a field portable hyperspectral imager capable of detecting micro-plastic contamination in soils for particle sizes between 0.5–5 mm. Whilst previous research has already successfully detected micro-plastic contamination using hyperspectral imaging [79], that study focused on micro-plastic detection within sea water filtrates, which required the manual separation of micro-plastics from the substrate prior to image acquisition due to difficulties related to plastic identification through water [78]. In contrast, the device developed by Shan et al. [78] enables in-situ measurements with minimal disruption to the study area. Given the increasing importance of this area, this technology is likely to be of ever increasing utility here in the future.

Furthermore, Chennu et al. [80] discuss the development of a diver-operated underwater device for the monitoring of shallow marine ecosystems, such as coral reefs. This device is the first of its kind and represents a significant, cost-effective improvement in hyperspectral data acquisition for these environments, avoiding the effects of complex optical paths through the atmosphere and the water column [80], associated with observations taken above the water surface. Whilst the spatial resolution of this sensor was lower than that of comparable digital camera imagers, it could sufficiently identify the spectral reflectance features of corals at the organism level. The user-friendly nature of this device allowed it to be operated with no prerequisite skills, however, its present design is too large for integration with unmanned platforms, highlighting a significant avenue for future research. 

The examples above highlight the versatility of these devices, with miniaturized hyperspectral sensors replacing conventional non-imaging spectroscopy in a number of application areas [14,47]. Furthermore, this proliferation appears set to continue as the speed of image capture, and the processing power of these units, increase year on year, just as the unit costs are reduced on an annual basis [47]. However, the development of more robust low-cost, field portable sensors for deployment in more extreme settings, e.g., glacial and volcanic regions, remains somewhat limited. The development of future low-cost hyperspectral sensors for these environments would build on the implementation of low-cost spectral technologies in hostile environments [39,81,82,83], which have been based in configurations suitable for short-term deployments. Indeed, Wilkes et al. [81] intimate that more sustained deployments would require significant improvements to the outer casings of the device for ruggedization and weatherproofing and robust product testing. This is a difficult hurdle to overcome due to the highly dynamic and often volatile nature of these environments, making year-round field-based monitoring challenging, even with state-of-the-art designs [62,84]. Future work could, therefore, involve improvement of robust low-cost hyperspectral imagers to allow them to successfully compete with their scientific grade equivalents for prolonged data collection in these more extreme environments. In this respect, UAV based units have the advantage that deployments are by nature discrete and time limited, rather than continuous, as discussed above.

## 5. Discussion

**Figure 4:**
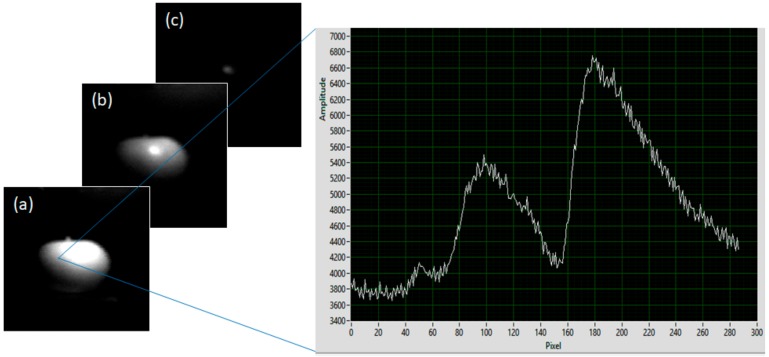
Example dataset captured using a low-cost hyperspectral device; 128 × 128 hyperspectral image displaying spectral reflectance from 340–850 nm of a green apple and tungsten filament lamp. Image tiles display reflectance peaks across the Red (**a**), Green (**b**), and Blue (**c**) portions of the electromagnetic spectrum. Note the corresponding peaks in reflectance captured in the spectral response graph.

The development of these devices, and their application to a panoply of environmental monitoring areas, represent a series of significant technical and scientific advances. These units provide accurate, high resolution datasets, which help to bridge the gap between sparse and discontinuous field observations and continuous but coarse resolution spaceborne technologies [14,15,62], as well as enabling real time analysis and decision making in environmental monitoring contexts [48], making them a beneficial addition to existing field monitoring techniques. Furthermore, miniaturized, low-cost systems can be operated on a local scale by small organizations and/or companies, considerably reducing the time required to organize specific remote sensing campaigns [14], relative to manned airborne surveys, reducing the need for expensive and time-consuming direct measurement methods and enabling affordable and rapid environmental monitoring [14]. This is particularly advantageous in less well-resourced countries, where there are acute needs in terms of crop monitoring, for example. However, there remain a number of limitations on these devices at present [40]. For UAV based applications, these limitations are largely related to the currently rather large weight, bulk, and power supply requirements of the deployed sensors, highlighting the need for future miniaturization in such devices. [15]. Although this hurdle is beginning to be overcome [42], often with the application of off-the-shelf consumer electronics components [47,50], there still typically remains a trade-off between sensor size and data quality in these next generation units [40,77,78]. Similar limitations also affect ground-based and hand-held devices, although in these contexts the restrictions are not as profound. The foremost challenge faced by the majority of these devices is their successful deployment for long-term data collection. However, with potential future developments in ruggedization of the hardware, which will allow such units to become competitive with commercial scientific grade devices for long-term field deployments, the application of ground-based hyperspectral imaging appears set to proliferate rapidly in the coming years (Figure 4).

With the technological move towards more compact, miniaturized devices for optical sensing [85,86], the implementation of low-cost consumer electronics in environmental monitoring is on the rise [85,86]. The application of smartphone-based spectroscopy has been of particular interest for a variety of disciplines [12,39,87], and is a technological step towards the realization of smartphone based hyperspectral imaging. Compared to basic mobile devices, smartphones are equipped with a number of features that expedite sensing applications [85], enabling performance of advanced scientific measurements [88,89]. This is particularly driven by the low-cost of these units, relative to commercial scientific grade cameras [86,90,91,92], resulting in these units being developed into a variety of lab-in-a-phone technologies [39,81,92,93]. Initial developments in this field have seen the creation of devices that work within the set-up of an existing smartphone, with considerable potential for future device development. However, current work has faced issues in connection with the unit operating systems, wherein raw data files (required for quantitative sensing applications) can be difficult to access and/or are effected by auto-scaling, e.g., Smith et al. [94], and the presence of Bayer filters within the majority of smartphone camera sensor designs, limiting most smartphone sensing to the visible portion of the electromagnetic spectrum within the three defined spectral bands corresponding to the cameras RGB pixels [82]. However, as smartphone-based spectrometers improve in performance, producing results similar to those of commercial scientific devices [39], the “compromise” in using these cheaper units, is becoming less of a relative downside. An in-depth review of these initial developments in smartphone spectroscopy can be found in McGonigle et al. [82].

As smartphone spectroscopy continues to develop, there is now the beginning of applying these units for hyperspectral imaging. In particular, Wilcox et al. [12], present an ultra-compact hyperspectral imaging system, for use within a UAV set-up, that has been developed to incorporate smartphone technologies. Similarly, Rissaren et al. [95], and Näsilä et al. [96], report initial developments in smartphone compatible hyperspectral imaging. Critically, this demonstrates that the ever increasing processor performance from state-of-the-art smartphone handsets is sufficient to manage the significantly larger data volumes associated with hyperspectral imaging in contrast to mere spectral data capture [10,12]. Just as smartphone spectroscopy has now been proven in a number of application areas [87,92,94], allowing for increased data collection at costs up to an order of magnitude lower than from conventional devices [81], it is likely that hyperspectral imaging with smartphones will be increasingly applied in the coming years.

In considering field portable hyperspectral imaging instrumentation for the majority of environmental monitoring settings, three design considerations are particularly pertinent:

- Compact light-weight design—Allowing for easy portability to a variety of field sites of varying accessibility. This criterion has particular benefits in relation to set-up times, enabling for rapid deployment of technical devices as well as significantly reducing the personnel requirements of field visits. As discussed above, this design feature is also of significant importance for sensors designed for UAV integration.
- Low-cost—Whilst this is not an essential requirement for successful environmental monitoring using field-based hyperspectral imaging, the production of low-cost sensors will increase the accessibility of this measurement modality, beyond the relatively limited field deployments achieved hitherto with the rather expensive previously available instrumentation. This is particularly the case for smartphone based platforms, given the ubiquity of these units, and their suitability for implementation as nodes within internet of things type architectures.
- Flexibility—In order to achieve the best results, deployed devices need to be easily configurable by researchers, allowing for adaptations to be made relating to the proposed device application. This criterion is most easily met by devices assembled “in house” as it allows researchers to develop and assemble components in the best arrangement for the proposed application, resulting in a device specifically designed for its task. This is typically more favourable than generic, commercial devices, which can be rather difficult to align to specific applications. Furthermore, a device developed “in house” can also provide significant reductions in set-up times as the researchers will generally be familiar with the device design.

Indeed, given the above it is evident that more and more research groups are opting to develop their own devices instead of relying on commercially available more expensive equipment, pointing to the proliferation and democratisation of hyperspectral imaging across the environmental sciences. Although, at present, many of these technologies are restricted by the current limitations of miniaturisation, and the associated tradeoffs that miniaturisation can bring in terms of the sensor performance, initial results from smartphone based hyperspectral imaging suggest that significant improvements in cost-effective, high spatial resolution data acquisition can be expected in the near future. This increase in performance, coupled to further reductions in instrumental cost, are likely to lead to increased utility and proliferation of these units in the coming decades, therefore.

However, an important additional consideration are the potential costs of required components external to the sensor design. This is of particular importance for sensors designed with the low-cost criterion in mind as the savings made during sensor assembly can quickly be lost through other device requirements. For example, when considering UAV integrated hyperspectral sensors, it is imperative that low-cost designs also adhere to the compact light-weight criterion in order to prevent the incursion of extensive costs related to the acquisition of UAVs with higher payload weight limits. As failure to consider this factor can lead to significant additional build costs, it is, therefore, of considerable importance to understand the payload specifications and limitations of the proposed UAV system in tandem with implementing the sensor design and development process. A number of articles discuss the variations, and subsequent categorisation, of different UAV systems, highlighting the, often substantial, differences in terms of payload weight, fuel requirements, and survey length [49,97,98,99]. In general, multi-rotor UAVs are more suited to operation within more confined/inaccessible field sites due to their ability to take off/land vertically, whereas fixed wing UAVs are typically suited to longer endurance applications and provide more stable data collection [96], however, the final decision as to which design of UAV is selected is established by the specific parameters of the proposed application and, therefore, varies substantially between projects. Nevertheless, these characteristics are of considerable importance to the successful deployment of a UAV integrated sensor and can significantly impact the overall cost to deliver the measurement. Furthermore, costs and payload weights can be minimized further with the thorough selection of required ancillary sensors, such as RGB cameras and GPS units, e.g., both Näsi et al. [43], and Honkavaara et al. [55], reduced the overall costs of their set-ups with the inclusion of additional small consumer cameras instead of more expensive top-of-the-range models. 

It is clear that in order to design a successful low-cost compact hyperspectral imaging instrument a complex list of design variables must be considered and potentially juggled to enable best delivery against the monitoring objectives. Within this there are two key exciting new frontiers, which these low-cost units now expedite: firstly, their potential for deployment and monitoring in less well-resourced countries, allowing for valuable research data to be acquired without the associated costs. Secondly there is the potential for future, long-term deployments in more extreme environments, for example with applicability in pioneering cost-effective early warning/monitoring systems for more volatile settings. Although the effectiveness of these units is limited by currently available technologies, the increasing interest and development in this sector looks set to produce vast improvements to low-cost and miniaturised hyperspectral data collection, and thus provides the opportunity to improve data sets across a wealth of environmental monitoring domains.

## 6. Conclusions

This article has provided an in-depth review of current miniaturized and low-cost field deployable hyperspectral technologies and their integration into the environmental monitoring field. Whilst the miniaturization and deployment of these devices is ongoing, it is evident that this is a burgeoning area of research with the potential to revolutionise environmental monitoring in a wide variety of fields, hence the timeliness of capturing the state-of-the-art, in this article, at this point in time. At present, these devices largely complement existing monitoring techniques, however, as technologies continue to improve, it is likely that they will be increasingly applied in stand-alone monitoring capacities. Future work should look to expanding the applications for these devices, in particular allowing them to be successfully utilized even in more extreme environments, as well as further capitalizing on the reduced cost of consumer available technology in this domain. With the latest low-cost devices now producing scientific grade results, it appears as though hyperspectral imaging with smartphones in particular is now set to become a promising new frontier in empirical environmental science, significantly broadening the reach of hyperspectral image capture. This article captures the beginning of what we anticipate will be a steep rising curve of community uptake, broadening applicability far beyond those application domains covered to date.
